# L-theanine promotes angiogenesis in limb ischemic mice by modulating NRP1/VEGFR2 signaling

**DOI:** 10.17305/bb.2024.11256

**Published:** 2025-01-07

**Authors:** Jingyi Wang, Yinghui Xu, Yating Ruan, Xinyang Hu

**Affiliations:** 1 Department of Cardiology of the Second Affiliated Hospital, School of Medicine, Zhejiang University, Hangzhou, China; 2 Zhejiang Cancer Hospital, Hangzhou, China; 3 State Key Laboratory of Transvascular Implantation Devices, Hangzhou, China; 4 Cardiovascular Key Laboratory of Zhejiang Province, Hangzhou, China

**Keywords:** Peripheral artery disease, PAD, angiogenesis, L-theanine, neuropilin-1, NRP1, vascular endothelial growth factor receptor 2, VEGFR2, NRP1/VEGFR2 signaling pathway

## Abstract

Peripheral artery disease (PAD), primarily caused by atherosclerosis, leads to the narrowing or blockage of arteries that supply blood to the limbs. This study explores the pro-angiogenic effects of L-theanine and its underlying mechanisms in a mouse model of hindlimb ischemia (HLI). To evaluate L-theanine’s pro-angiogenic effects, human umbilical vein endothelial cells (HUVECs) were subjected to tube formation, migration, sprouting, and proliferation assays. *In vivo*, C57BL/6 mice with induced HLI were treated with L-theanine. Blood flow recovery was measured via Doppler ultrasound, and vascular density was analyzed using immunofluorescence staining. RNA sequencing identified neuropilin-1 (NRP1) as a key regulator, and the expression levels of NRP1 and VEGFR2 were examined through qPCR and Western blotting. L-theanine significantly enhanced angiogenesis in HUVECs, as demonstrated by improved tube formation, migration, sprouting, and proliferation. In mice, L-theanine treatment resulted in increased vessel density and improved blood flow recovery. Furthermore, L-theanine was found to activate the NRP1/VEGFR2 signaling pathway in both HUVECs and the HLI mouse model. These findings indicate that L-theanine can promote angiogenesis and activate key pathways involved in vascular repair, suggesting its potential as a therapeutic agent for treating vascular defects associated with PAD.

## Introduction

Peripheral artery diseases (PADs) encompass a range of conditions affecting blood vessels outside the heart and brain [1, 2]. These diseases pose significant healthcare challenges due to their high prevalence, diverse etiologies, and potentially severe consequences [3, 4]. Traditional therapeutic approaches primarily rely on antiplatelet agents to prevent thrombosis and lipid-lowering drugs to manage atherosclerosis [5, 6]. However, these treatments often fail to directly address the fundamental issue of impaired blood flow caused by narrowed or occluded vessels [7, 8]. Consequently, there is a pressing need to explore novel therapeutic strategies that promote angiogenesis to restore blood flow. Angiogenesis, the formation of new blood vessels from pre-existing ones, is a vital physiological process essential for tissue growth, repair, and regeneration [9, 10]. This mechanism plays a crucial role in various biological contexts, including embryonic development, wound healing, and the remodeling of tissues affected by ischemic diseases [11, 12].

L-theanine, chemically known as N-ethyl-L-glutamine, is an amino acid derivative predominantly found in tea leaves, particularly in Camellia sinensis [13]. It possesses a unique chemical structure characterized by an ethylamide group attached to the gamma (γ) position of the glutamic acid backbone [14]. Its chemical stability and ability to cross the blood–brain barrier have made L-theanine a compound of interest in neurological research, as demonstrated in various studies [15–17]. These properties enable it to exert a range of physiological effects, including the modulation of immunity [18], enhancement of cognitive function [19, 20], and antioxidant activity [21]. However, its role in angiogenesis remains underexplored, presenting a novel avenue for investigation. Neuropilin-1 (NRP1) and vascular endothelial growth factor receptor 2 (VEGFR2) are pivotal regulators of angiogenesis [22]. When vascular endothelial growth factor (VEGF) binds to VEGFR2, it initiates a cascade of intracellular signaling pathways that drive endothelial cell processes critical for new blood vessel formation [23, 24]. NRP1, acting as a co-receptor for VEGF, enhances VEGFR2 signaling by increasing its activity and stability [22]. This interaction between NRP1 and VEGFR2 is essential for optimizing the angiogenic response and ensuring effective blood vessel development. Consequently, the NRP1/VEGFR2 axis has emerged as a key therapeutic target for enhancing angiogenesis. Advances in this area hold significant promise for improving patient outcomes by facilitating tissue recovery and restoring function in conditions characterized by insufficient blood supply [25]. In this study, we investigated the effects of L-theanine (1 mM) on human umbilical vein endothelial cells (HUVECs) *in vitro* and observed that it significantly enhanced tube formation, migration, and proliferation. Furthermore, L-theanine treatment improved blood flow restoration and increased blood vessel density in the hindlimb ischemia (HLI) model *in vivo*. Mechanistically, L-theanine was shown to elevate NRP1 and VEGFR2 levels, thereby activating the AKT signaling pathway. Notably, silencing NRP1 after L-theanine treatment reduced angiogenesis, confirming L-theanine’s role as a regulator of the NRP1/VEGFR2 pathway. These findings provide valuable insights into the potential of L-theanine as a novel therapeutic agent for ischemic limb conditions, potentially paving the way for new targeted strategies in angiogenesis research.

## Materials and methods

### Chemicals

L-theanine (98% purity) was purchased from Sigma-Aldrich (Cat# SMB00395), dissolved in PBS, and stored as a stock solution in aliquots at −20 ^∘^C. Additional chemicals and reagents are listed in Table S1.

### Cell cultures

HUVECs were obtained from ATCC (Cat#PCS-100-013) and cultured on dishes coated with 0.1% gelatin in M199 medium. The medium contained 1 mg/mL D-glucose, 20% fetal bovine serum (FBS), 2 mM L-glutamine, 30 mg/L endothelial cell growth factor supplements (EGCS), 10 units/mL heparin, 50 IU/mL penicillin, and 50 µg/mL streptomycin. Cells at passages 1–5 were used and maintained in a 5% CO_2_ incubator at 37 ^∘^C. HUVECs were stimulated with 1 mM L-theanine for 12 h *in vitro.*

### Proliferation assay

The assessment of cell proliferation was conducted using the CCK-8 assay. In brief, HUVECs were seeded into 96-well plates at a density of 3 × 10^3^ cells per well. Each time point included six replicates per group. At 3, 24, 48, and 72 h, 10 µL of CCK-8 solution was added to each well, followed by a 2-h incubation. Afterward, absorbance was measured at 450 nm.

### Tube formation assay

To prepare the 96-well plates, 50 µL of growth factor-reduced Matrigel was applied to each well, followed by a 30-min incubation at 37 ^∘^C. Afterward, 20,000 endothelial cells (ECs) were seeded per well in 50 µL of Matrigel-containing medium. L-theanine (1 mM) was added to the culture media as specified. Following a 12-h incubation period, tube-like structures were visualized using light microscopy (5× magnification, Leica DM3000). The total tube length was quantified using Image-Pro Plus software.

### Transwell assay

HUVECs were cultured in a medium containing 1% FBS for overnight starvation. Subsequently, 2.5 × 10^ImEquation2^ cells were seeded into the upper compartment using EBM-2 medium supplemented with 1% FBS. Freshly prepared EBM-2 medium containing 10% FBS was added to the lower well. ECs were allowed to migrate for 24 h before being fixed in 4% paraformaldehyde solution overnight. The cells were then stained with a 1% crystal violet solution for 10 min. Migrated cells were counted using a light microscope at 5.0× magnification (Leica DM3000). For each well, three random fields were analyzed, and the mean cell count was calculated for each biological replicate.

### Spheroid sprouting assay

HUVECs were suspended in EGM-2 medium containing 20% methylcellulose and incubated overnight in hanging drops to form spheroids. The spheroids were subsequently embedded in collagen gel and cultured for 20 h to induce sprouting. Cultures were fixed with 4% PFA at room temperature and imaged under light microscopy (10×, Leica DM3000). The number of sprouts per spheroid and the total sprout length (the cumulative length of sprouts and branches per spheroid) were analyzed using Fiji/ImageJ software.

### Immunoblotting

HUVECs pre-starved for 30 min were stimulated with 50 ng/mL VEGF (VEGF Recombinant Mouse Protein, Life Technologies, Cat# PMG0114) for 5 min to detect the mechanism of action. Prepare 8%–10% SDS-PAGE gels at the desired concentration and assemble the PAGE apparatus with fresh running buffer. Perform electrophoresis at a constant current of 20–30 mA per plate until the bromophenol blue dye reaches the bottom of the gel, indicating the process is complete. Next, prepare fresh transfer buffer and cool it at 4 ^∘^C. Activate the PVDF membrane (Millipore, USA) by soaking it in methanol. Transfer the proteins from the gel to the PVDF membrane using a constant current of 250 mA for approximately 2.5 h. Block the membranes with 5% non-fat milk in PBST (PBS containing 0.1% Tween-20) for 1 h at room temperature. Discard the blocking solution and wash the membrane four times with PBST. Incubate the membranes overnight at 4 ^∘^C with primary antibodies (CD31, NRP1, p-VEGFR2/t-VEGFR2, p-Akt/Akt) diluted at 1:1000, except for β-actin. After four washes with PBST, incubate with HRP-conjugated secondary antibodies for 2 h. Finally, wash the membrane three times with PBST before imaging using a Bio-Rad Protein Imager. Capture and quantify protein bands using Image Lab software. Refer to Supplementary Table S2 for details on the primary antibodies used.

### Quantitative RT-PCR (qRT-PCR) analysis

Total RNA was isolated using the TRIzol reagent (Invitrogen). RNA was then reverse-transcribed into cDNA using a reverse transcription kit and oligo (dT) primers. The resulting cDNA was diluted to a suitable concentration. PCR amplification was performed using the SYBR Green assay kit (Invitrogen, Catalog No. A25779) according to the following protocol: an initial denaturation at 95 ^∘^C for 3 min, followed by 40 cycles of 95 ^∘^C for 15 s and 60 ^∘^C for 30 s. Primers were designed based on sequences available in GenBank. The specific primer sequences are as follows: ID1 (Homo sapiens): F-GCACGTCATCGACTACATCAG, R-ACGCATGCCGCCTCGRCAN1 (Homo sapiens): F-GTATGAATTGCACGCAGCGA, R-CGGCCTCCTGGTCTGGATAIRS1 (Homo sapiens): F-ACTTGAGCTACGGTGACGTG, R-AGCTGATGGTCTTGCTGGTCPRKCD (Homo sapiens): F-TGACACTTGCCGCAGAGAAT, R-GGTAGAGTTCAAAGCGGCCTNRP1 (Homo sapiens): F-TGATGAAACAGGGAGCACGC, R-TGGTGATGAGGATGGGGTCTTFPI2 (Homo sapiens): F-GAGATCTGTCTCCTGCCCCT, R-TAGAAATTGTTGGCGTTGCCCLPAR1 (Homo sapiens): F-CTCGGCATAGTTCTGGACCC, R-CTGTGGACAGCACACGTCTAEPOR (Homo sapiens): F-TACCCCACCCCACCTAAAGT, R-CATCGGATAAGCCCCCTTGGMMP15 (Homo sapiens): F-CTAAAGGGGCCTTCCTGAGC, R-GCAGGATGGACTTGGGGTAGCDC25C (Homo sapiens): F-ACCTGCTCCTGGAGAGAGAC, R-GCAACGTTTTGGGGTTCCTCSOX4 (Homo sapiens): F-ACCTGAACCCCAGCTCAAAC, R-CAGTAGTCCGGGAACTCGAAGHDAC9 (Homo sapiens): F-AGCCTGACCTCATGTGGAAC, R-CTGTGCATTCTTTGCTGAGCC. The primers amplify fragments that are 100–300 bp in length. Data analysis was conducted using the Roche LightCycler 480 II software to determine the threshold cycle (Ct) values. The Ct values were normalized to β-ACTIN expression levels, and relative gene expression was calculated using the 2^−ΔΔCt^ method. Detailed primer sequences are listed in Table S3.

### siRNA transfection experiments

To silence NRP1 expression, siRNA targeting NRP1 (purchased from Shanghai Genechem, China) was used. The target sequences were as follows: sense (5′-3′): GAAGGUUUCUCAGCAAACUTT; antisense (5′-3′): AGUUUGCUGAGAAACCUUCTT. Cells were plated in ECM and allowed to reach 50%–70% confluency. Transfection complexes were prepared by diluting siRNAs in serum-free medium and mixing them with the transfection reagent. The complexes were incubated for 15–30 min at room temperature to facilitate complex formation. These transfection complexes were then added to the cells. Following transfection, the cells were incubated at 37 ^∘^C in 5% CO_2_ for 6 h. The medium was then replaced with serum-containing growth medium, and the cells were incubated for 48 h before conducting knockdown experiments. To confirm NRP1 knockdown, gene silencing efficiency was validated using qRT-PCR and Western blot analysis.

### Murine HLI model with L-theanine or shNRP1 administration

To maintain body temperature, 10-week-old C57 mice were anesthetized intraperitoneally (i.p.) with ketamine/xylazine (80/5 mg/kg) and kept warm using a heated blanket. The distal portion of the saphenous artery and the proximal portion of the femoral artery were ligated using 7–0 sutures. All arteries in the intermediate space were excised, and branches between the two ligated locations were also tied off. Hindlimb blood flow was immediately assessed after inducing HLI using noninvasive laser Doppler imaging. Follow-up imaging was conducted on days 3, 7, and 14 post-HLI. For *in vivo* studies involving L-theanine, 100 µL of a 1 mg/kg L-theanine solution was administered intraperitoneally to the rodents one day prior to HLI, as well as on days 1, 5, and 10 post-surgery. In addition, mice received intramuscular injections of 1 × 10^9^ PFU adeno-associated virus (AAV; NC or shNRP1) in 10 µL of DMEM per mouse via microinjector, administered four weeks before HLI. The AAV-shNRP1 was purchased from Shanghai Genechem, China (target sequences: ACAGCTTGGAGTGCACCTACA).

### Immunofluorescence staining

The freshly collected whole gastrocnemius muscle samples were embedded in O.C.T. compound, submerged in isopentane, and rapidly cooled with liquid nitrogen. The frozen tissue sections were fixed with 10% formaldehyde for 8 h and subsequently permeabilized using 0.5% Triton X-100 in PBS. After blocking with a PBS solution containing 10% bovine serum albumin, the sections were incubated overnight at 4 ^∘^C with primary antibodies, followed by incubation with the corresponding secondary antibodies. Finally, the nuclei were counterstained with DAPI.

### RNA sequencing (RNA-seq)

Collect HUVEC samples from the PBS and L-theanine groups. Extract total RNA using the TRIzol reagent, and assess both the quality and quantity of the extracted RNA. Enrich mRNA from the total RNA pool, then convert the enriched mRNA into a cDNA library suitable for sequencing. Perform high-throughput sequencing on the prepared RNA libraries to generate raw sequencing data. Process the raw sequencing data by performing quality control, aligning reads to a reference genome or transcriptome, quantifying gene expression levels, and identifying differentially expressed genes. Expression values (FPKM; Fragments Per Kilobase of transcript per Million mapped reads) were calculated for each gene, and differentially expressed genes (with a false discovery rate (FDR) of < 0.05) were identified using the Cufflinks Assembly and Differential Expression v1.1.0 application. Equal amounts of RNA from different groups (*n* ═ 3) at the starting point were used for normalization. Finally, perform functional annotation and pathway analysis of the differentially expressed genes to gain insights into the relevant biological processes and pathways.

### Ethical statement

All live animal experiments were conducted in strict compliance with the ARRIVE guidelines. These experiments were approved by the Institutional Animal Care and Use Committee of Zhejiang University School of Medicine’s Second Affiliated Hospital (Approval Number: 2024224). Male C57BL/6J mice were obtained from Shanghai Slac Laboratory Animal Technology Corporation. The mice were maintained on a standard laboratory diet and housed under a 12-h light/dark cycle. For anesthesia, the mice were intraperitoneally administered ketamine (80 mg/kg) and xylazine (5 mg/kg) and placed on a heated mat to maintain a constant temperature. Their physiological parameters—including heart rate, respiration rate, and body temperature—were continuously monitored using a non-invasive blood oxygen monitor to ensure their well-being. Efforts were made to minimize the number of animals used and to alleviate their suffering throughout the experimental process.

### Statistical analysis

GraphPad Prism 7 was used to analyze the statistical significance of the data and to generate graphical representations. The statistical test used to determine significance for each specific experiment is indicated in the corresponding figure legend. For comparisons between two groups, an unpaired *t*-test was conducted. When more than two groups were involved, a one-way or two-way ANOVA was used, followed by Bartlett’s test to assess differences. Prior to performing these statistical tests, the Shapiro–Wilk normality test was used to evaluate data distribution and confirm whether it followed a normal distribution. All statistical analyses were conducted using GraphPad Prism 7 (*P < 0.05, **P < 0.01, and ***P < 0.001). Data are presented as mean ± SEM for all experiments.

## Results

### Investigation of L-theanine with pro-angiogenic effects *in vitro*

**Figure 1.**
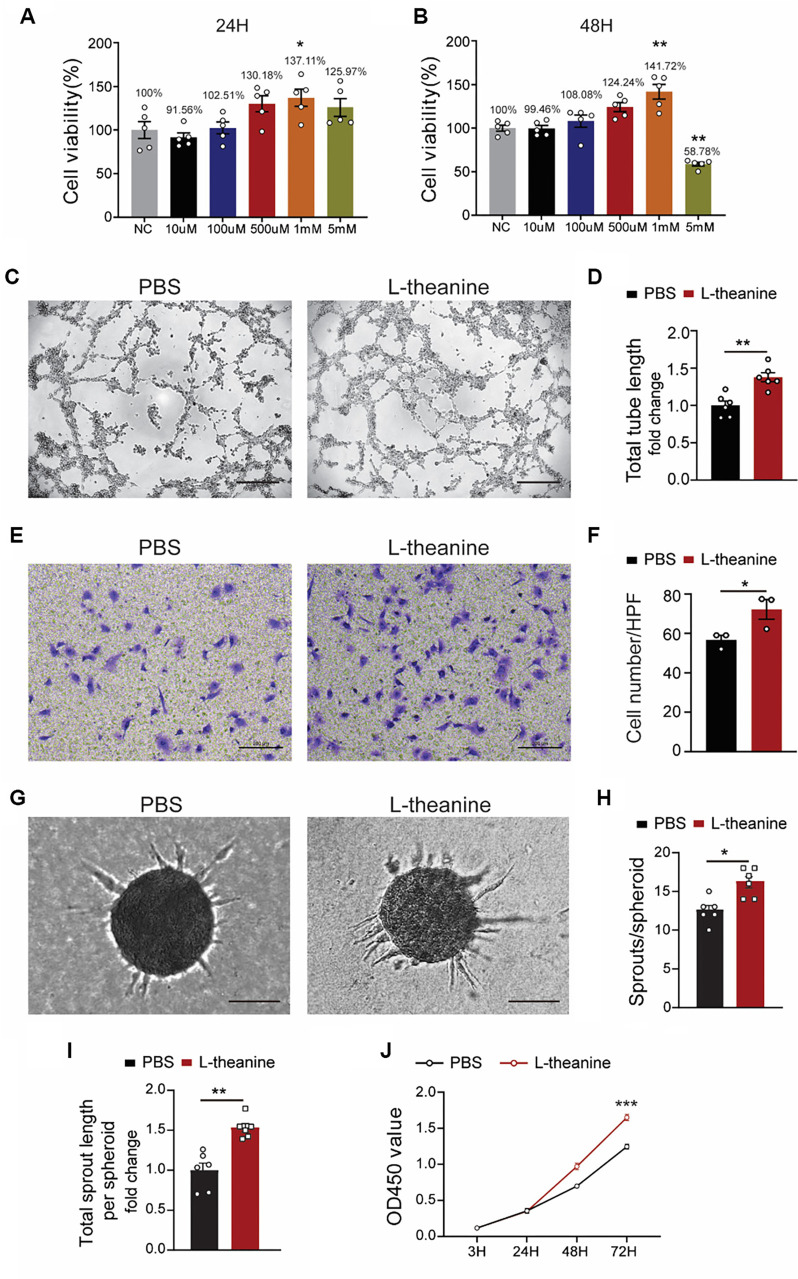
**L-theanine promotes the angiogenesis of HUVECs *in vitro*.** (A and B) Quantification of OD450 values of HUVECs in different concentration of L-theanine at different time points (one-way ANOVA, 24 h: *P* ═ 0.0124; and 48 h: NC vs 1 mM *P* ═ 0.002 NC vs 5mM *P* ═ 0.002); (C and D) Images and total tube length of tube networks formed by HUVECs in tube formation assay (*n* ═ 6; *t*-test, *P* ═ 0.0013); (E and F) Images and the number of migrated HUVECs per HPF in Transwell assay (*n* ═ 6; *t*-test, *P* ═ 0.0481); (G–I) Images, number of sprouts per spheroid, and total sprout length of spheroids (*n* ═ 6; *t*-test; *P* ═ 0.0492); (j) Quantification of OD450 values of HUVECs in PBS and L-theanine groups at different time points (3, 24, 48, and 72 h; two-way ANOVA, *P* < 0.001). Quantified data are presented as means ± SEM.

To evaluate the effect of L-theanine on angiogenesis, we initially treated HUVECs with various concentrations of L-theanine dissolved in PBS and assessed cell viability at 24 and 48 h. The results showed that cell survival was enhanced at a treatment concentration of 1 mM L-theanine (Figure 1A and 1B). Based on these findings, we selected 1 mM L-theanine for further experiments, with PBS serving as the control. A tube formation assay demonstrated that L-theanine treatment significantly enhanced the vessel formation capacity of HUVECs compared to controls (Figure 1C and 1D). Similarly, the Transwell assay revealed that L-theanine increased the migratory ability of HUVECs relative to the control group (Figure 1E and 1F). Additionally, L-theanine was found to increase sprouting (Figure 1G and 1I) and proliferation (Figure 1J) in HUVECs compared to controls. Collectively, these data confirm that L-theanine promotes angiogenesis *in vitro.*

### L-Theanine exerts therapeutic angiogenic effect in HLI model

**Figure 2.**
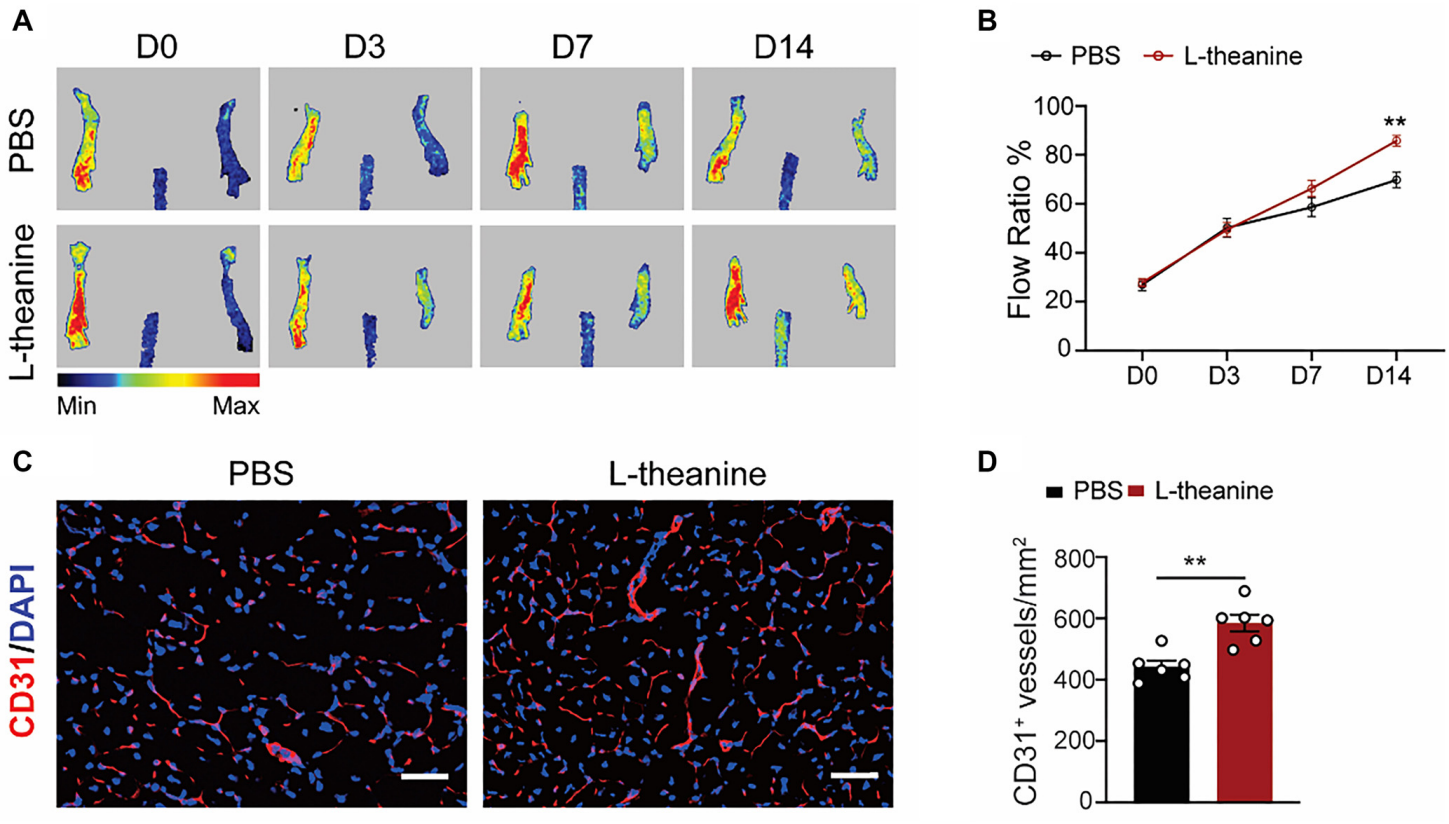
**L-theanine promotes angiogenesis in HLI model.** (A and B) Blood flow was evaluated via laser Doppler imaging on Day 0, Day 3, Day 7, and Day 14 after HLI, and quantified as the ratio of measurements of the injured (HLI) and uninjured (non-HLI) limbs (*n* ═ 6; two-way ANOVA, *P* ═ 0.0035); (C and D) Immunofluorescence staining for CD31 in gastrocnemius muscles. scale bar, 50 µm. Quantification of CD31 positive vessels per mm^2^ (*n* ═ 6; *t*-test, *P* ═ 0.0017). Quantified data are presented as means ± SEM. HLI: Hindlimb ischemia.

To explore the *in vivo* therapeutic potential of L-theanine under ischemic conditions, L-theanine (1 mg/kg) and the negative control (PBS) were administered intraperitoneally one day prior to the induction of HLI (D-1) and again on D1, D5, and D10 after the surgery. Doppler imaging was used to monitor hindlimb blood flow in the two groups of mice over a two-week period following surgery. The results demonstrated that exogenous L-theanine significantly improved hindlimb blood flow compared to the control group (Figure 2A and 2B). Furthermore, immunostaining revealed a notable increase in CD31+ vessels in the L-theanine-treated group relative to controls (Figure 2C and 2D). These findings indicate that L-theanine effectively promotes angiogenesis in ischemic limbs *in vivo.*

### L-theanine promotes NRP1 expression in HUVECs

**Figure 3.**
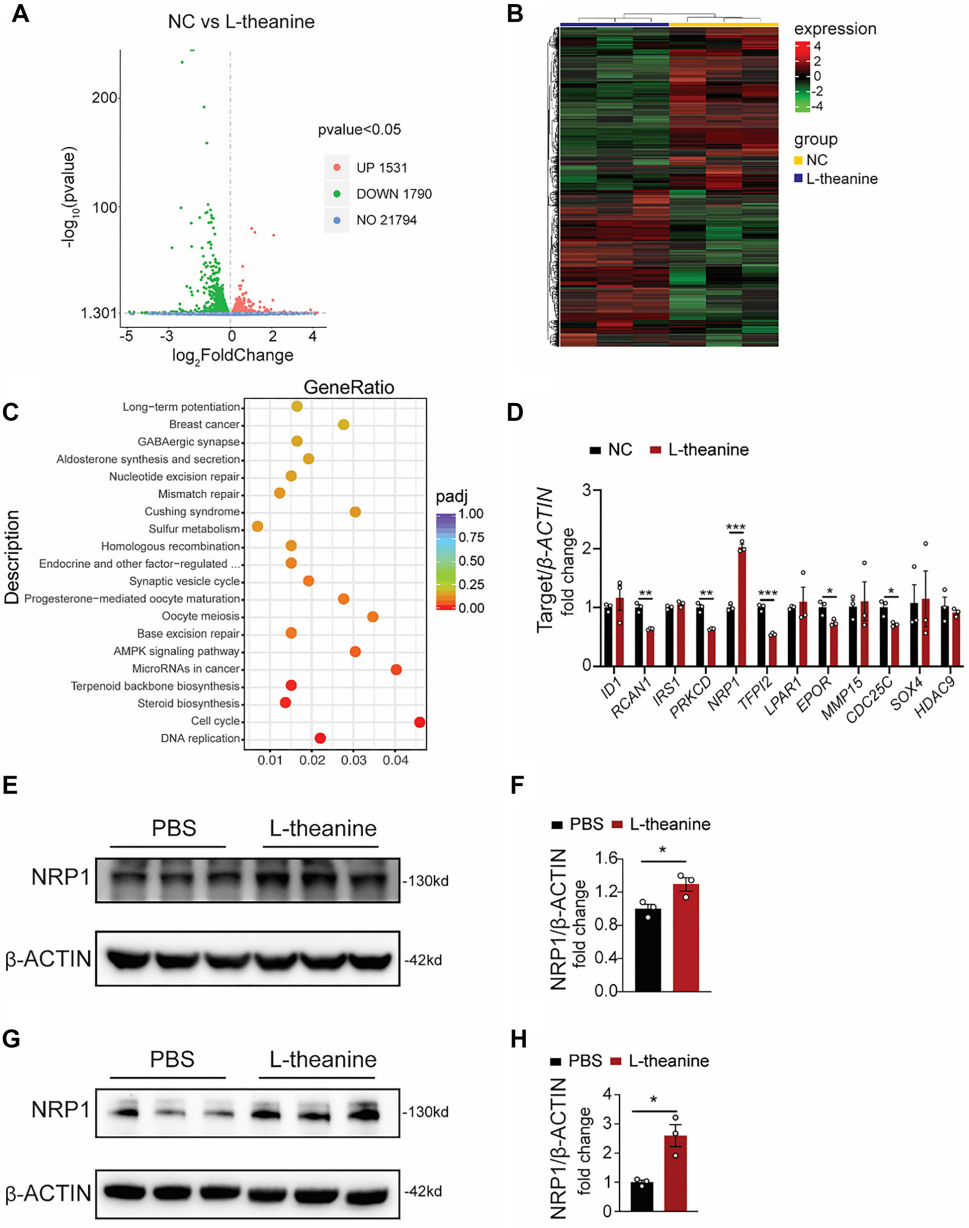
**L-theanine promotes *NRP1* expression in HUVECs.** (A) The RNA sequencing analysis on HUVECs from the PBS and L-theanine-treated groups. The volcano plot showed differentially expressed genes between the L-theanine group and the control group. Red points indicated upregulated genes, green points indicated downregulated genes, and grey points represented genes with no significant change. (B) The heatmap displayed the expression patterns of differentially expressed genes between the L-theanine group (blue) and the control group (yellow). (C) The KEGG pathway diagram depicted the pathways significantly affected by L-theanine treatment. (D) L-theanine treated group exhibited elevated mRNA levels of *NRP1* compared to the control group. (E and F) NRP1 protein levels in HUVECs with L-theanine or PBS treatment, and quantitative proteins were plotted, *n* ═ 3, *P* ═ 0.0223; (G and H) NRP1 protein levels detected in PBS and L-theanine treated mice after HLI, and quantitative proteins were plotted, *n* ═ 3, *P* ═ 0.0239; Quantified data are presented as means ± SEM, and significance was evaluated via a two-tailed unpaired *t*-test. NRP1: Neuropilin-1; HUVEC: Human umbilical vein endothelial cell.

To explore the potential mechanism by which L-theanine regulates angiogenesis, we performed bulk RNA sequencing analysis on HUVECs from both PBS- and L-theanine-treated groups. This analysis identified 1531 upregulated and 1790 downregulated genes in the L-theanine-treated group (Figure 3A and 3B). KEGG pathway analysis of the transcriptome data revealed significant activation of the cell cycle pathway following L-theanine treatment (Figure 3C). Among the significantly altered genes, 12 had been previously reported to play roles in vascular-related processes [22, 26–36]. Further qPCR analysis revealed that NRP1 was the most markedly upregulated gene in the L-theanine-treated group compared to the control group (Figure 3D). Protein-level verification via Western blotting confirmed a significant increase in NRP1 expression in HUVECs treated with L-theanine *in vitro* (Figure 3E and 3F). This trend was also observed *in vivo*, where administration of exogenous L-theanine in HLI models led to elevated NRP1 protein expression (Figure 3G and 3H). These findings demonstrate that L-theanine effectively modulates NRP1 expression under ischemic conditions.

### L-theanine upregulates the VEGFR2/AKT signaling by NRP1

**Figure 4.**
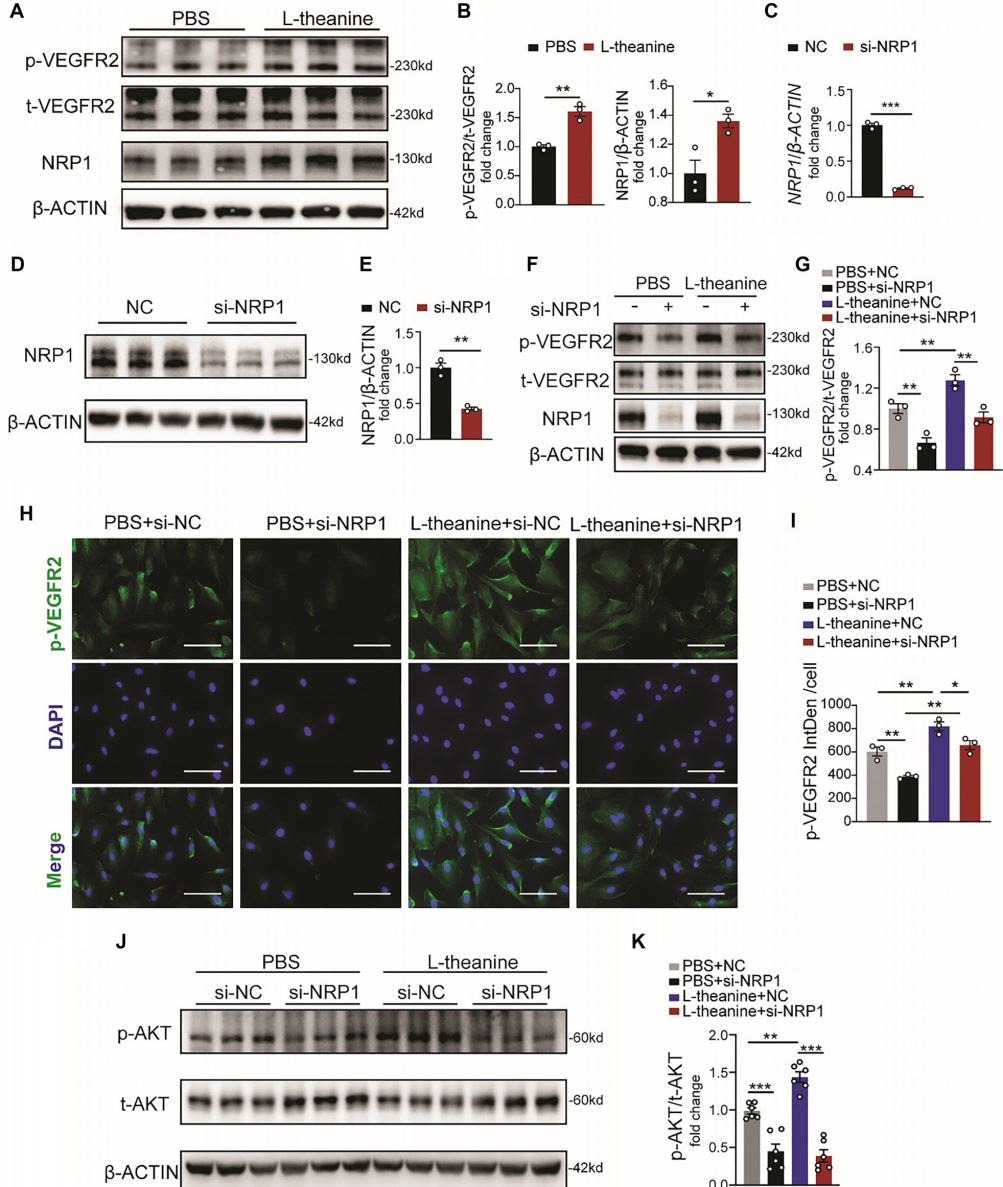
**L-theanine improves the VEGFR2/AKT signaling by increasing the expression of NRP1.** (A and B) The phosphorylation level of VEGFR2 in HUVECs treated with L-theanine or PBS, *n* ═ 3 (*t*-test; VEGFR2: *P* ═ 0.0023; NRP1: *P* ═ 0.0240); (C) The RNA level of *NRP1* after *NRP1* silenced, *n* ═ 3 (*t*-test, *P* < 0.001); (D and E) NRP1 protein levels detected in HUVECs that were subjected to treatment with si-NRP1 or NC, and quantitative proteins were plotted, *n* ═ 3 (*t*-test, *P* ═ 0.0011); (F and G) The phosphorylation and total level of VEGFR2 protein levels detected in *NRP1* or NC silenced HUVECs after treated with PBS or L-theanine as labeled, and quantitative proteins were plotted, *n* ═ 3 (one-way ANOVA; PBS+NC vs PBS+si-NRP1 *P* ═ 0.0082; PBS+NC vs L-theanine+NC *P* ═ 0.0084; L-theanine+NC vs L-theanine+si-NRP1 *P* ═ 0.0092); (H and I) The phosphorylation level of VEGFR2 detected through immunofluorescence staining (blue: DAPI; green: VEGFR2; scale bar, 100µm. *N* ═ 3; one-way ANOVA; PBS+NC vs PBS+si-NRP1 *P* ═ 0.0084; PBS+NC vs L-theanine+NC *P* ═ 0.0081; L-theanine+NC vs L-theanine+si-NRP1 *P* ═ 0.0395; PBS+si-NRP1 vs L-theanine+si-NRP1 *P* ═ 0.0020); (J and K) The phosphorylation and total level of AKT protein levels detected in *NRP1* or NC silenced HUVECs after treated with PBS or L-theanine as labeled, and quantitative proteins were plotted, *n* ═ 6 (one-way ANOVA; PBS+NC vs PBS+si-NRP1 *P* < 0.001; PBS+NC vs L-theanine+NC *P* ═ 0.0022; L-theanine+NC vs L-theanine+si-NRP1 *P* < 0.001). Quantified data are presented as means ± SEM. NRP1: Neuropilin-1; VEGFR2: Vascular endothelial growth factor receptor 2; HUVEC: Human umbilical vein endothelial cell.

NRP1 plays a critical role in angiogenesis by acting as a co-receptor for VEGFR2, significantly enhancing VEGFR2’s binding affinity to VEGF. This interaction leads to an upregulation of the downstream AKT pathway, thereby further promoting angiogenesis [37, 38]. In our study, we investigated whether L-theanine regulates the VEGFR2 signaling pathway via NRP1. Our results demonstrated that L-theanine treatment in HUVECs significantly increased VEGFR2 phosphorylation compared to the control group (Figure 4A and 4B). To further explore this mechanism, we used siRNA to suppress NRP1 expression (Figure 4C–4E). The knockdown of NRP1 caused a pronounced reduction in VEGFR2 phosphorylation in L-theanine-treated cells compared to controls (Figure 4F and 4G). Immunofluorescence staining confirmed this reduction, showing decreased VEGFR2 phosphorylation in L-theanine-treated cells following NRP1 silencing (Figure 4H and 4I). Moreover, NRP1 silencing also diminished the phosphorylation of the downstream AKT pathway (Figure 4J and 4K). Collectively, these findings suggest that L-theanine enhances VEGFR2/AKT signaling by upregulating NRP1 expression.

### L-theanine promotes angiogenesis through NRP1 *in vitro*

**Figure 5.**
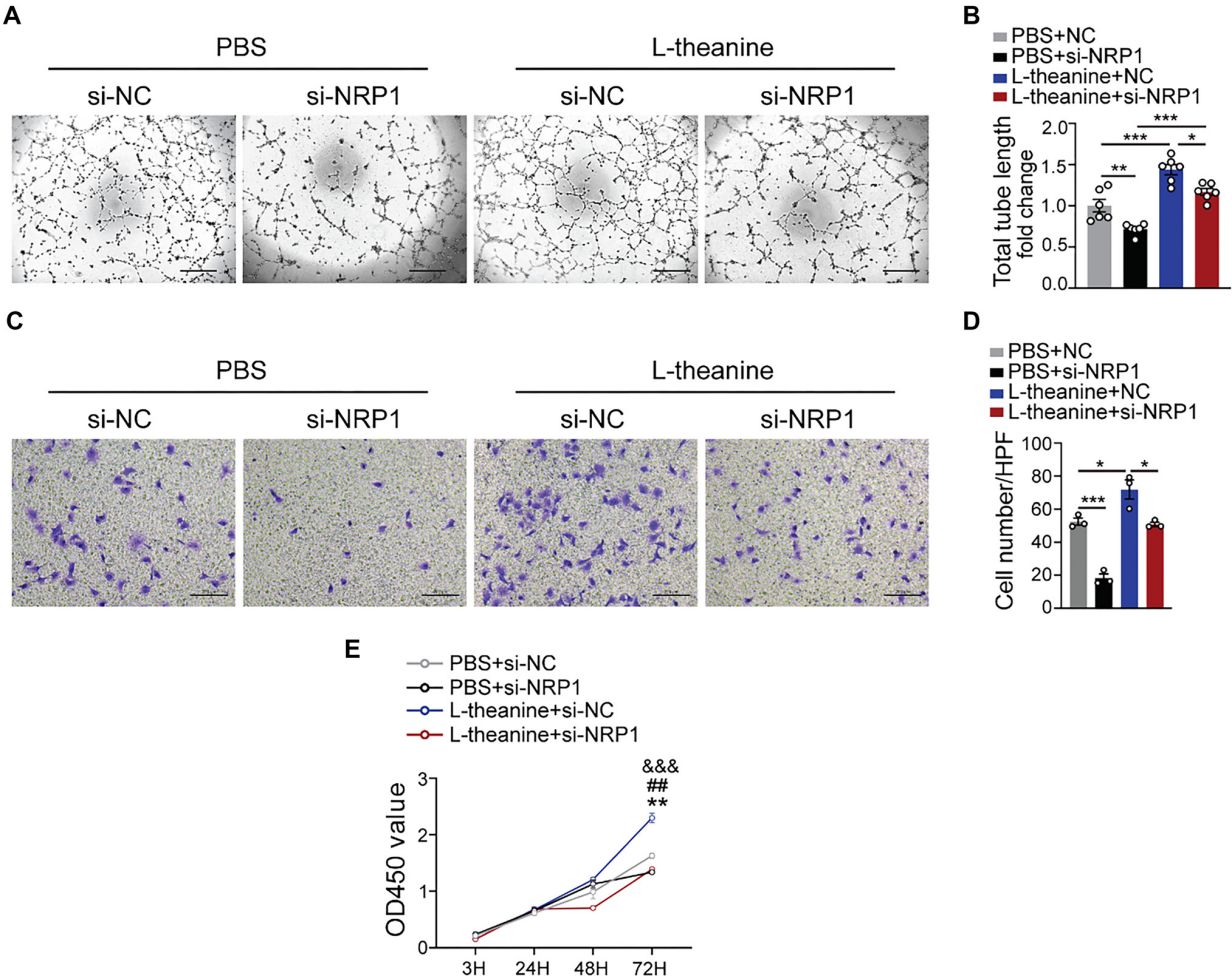
**L-theanine promotes angiogenesis *in vitro* through NRP1.** (A and B) Images and total tube length of tube networks formed by HUVECs (*n* ═ 6; one-way ANOVA; PBS+NC vs PBS+si-NRP1 *P* ═ 0.006; PBS+NC vs L-theanine+NC *P* < 0.001; L-theanine+NC vs L-theanine+si-NRP1 *P* ═ 0.032; PBS+si-NRP1 vs L-theanine+si-NRP1 *P* < 0.001); (C and D) Images and the number of migrated HUVECs per HPF (*n* ═ 6; one-way ANOVA; PBS+NC vs PBS+si-NRP1 *P* ═ 0.0005; PBS+NC vs L-theanine+NC *P* ═ 0.0163; L-theanine+NC vs L-theanine+si-NRP1 *P* ═ 0.0104); (E) Quantification of OD450 values at different time points (3, 24, 48, and 72 h). Quantified data are presented as means ± SEM, and significance was evaluated via two-way ANOVA (**P ═ 0.0096 PBS+si-NC vs PBS+si-NRP1; ^##^*P* ═ 0.0013, PBS+si-NC vs L-theanine+si-NC; and ^&&&^*P* < 0.001, L-theanine+si-NC vs L-theanine+si-NRP1). NRP1: Neuropilin-1.

Having established the pivotal role of NRP1 in L-theanine-mediated angiogenesis, we next examined the phenotypic changes resulting from NRP1 interference in HUVECs. Our experiments revealed that vessel formation capacity was significantly impaired in the L-theanine treatment group following NRP1 inactivation, as assessed via the tube formation assay (Figure 5A and 5B). Moreover, NRP1 silencing led to a pronounced reduction in migration within the L-theanine-treated group, as demonstrated by the Transwell assay (Figure 5C and 5D). Similarly, the proliferation of HUVECs was markedly reduced in the L-theanine-treated group when NRP1 was silenced, as shown by the CCK8 assay (Figure 5E). These findings provide compelling evidence that L-theanine promotes angiogenesis *in vitro* primarily through NRP1 activation.

### Pro-angiogenic effect of L-theanine in HLI models via NRP1

**Figure 6.**
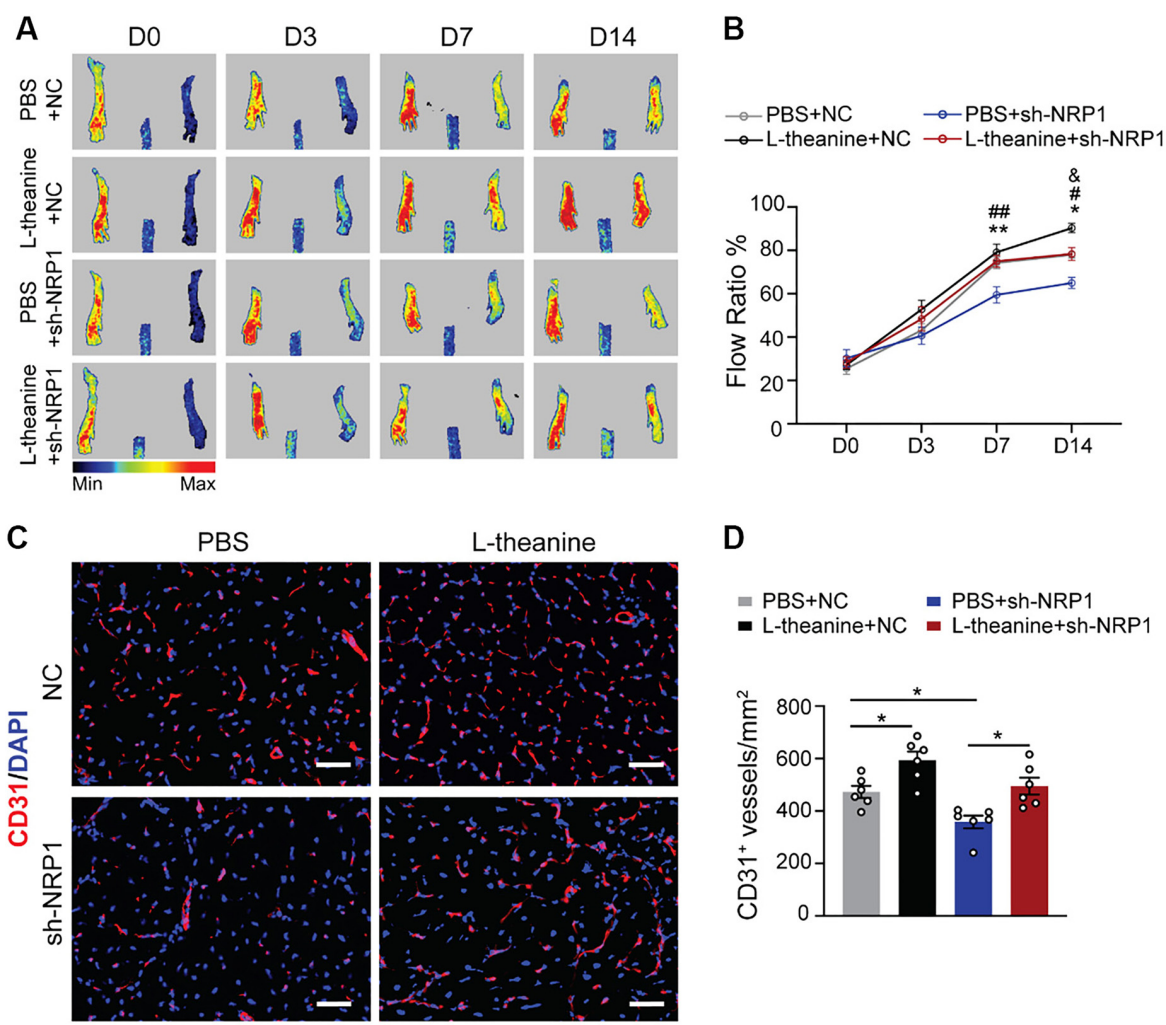
**L-theanine promotes angiogenesis in HLI models through NRP1.** (A and B) Blood flow was evaluated via laser Doppler imaging and quantified as the ratio of measurements of the injured (HLI) and uninjured (non-HLI) limbs (two-way ANOVA; D14: ^*^P ═ 0.0296, PBS+NC vs PBS+sh-NRP1; ^#^*P* ═ 0.0233, PBS+sh-NRP1 vs L-theanine+shNRP1. ^&^*P* ═ 0.0412, PBS+NC vs L-theanine+NC; *n* ═ 6); (C and D) Immunofluorescence staining for CD31 in gastrocnemius muscles. scale bar, 50 µm. Quantification of CD31 positive cells per mm^2^ (*n* ═ 6; PBS+NC vs PBS+sh-NRP1 *P* ═ 0.0458; PBS+sh-NRP1 vs L-theanine+shNRP1 *P* ═ 0.0143; PBS+NC vs L-theanine+NC *P* ═ 0.0331). Quantified data are presented as means ± SEM, and significance was evaluated via one-way ANOVA. HLI: Hindlimb ischemia; NRP1: Neuropilin-1.

**Figure 7.**
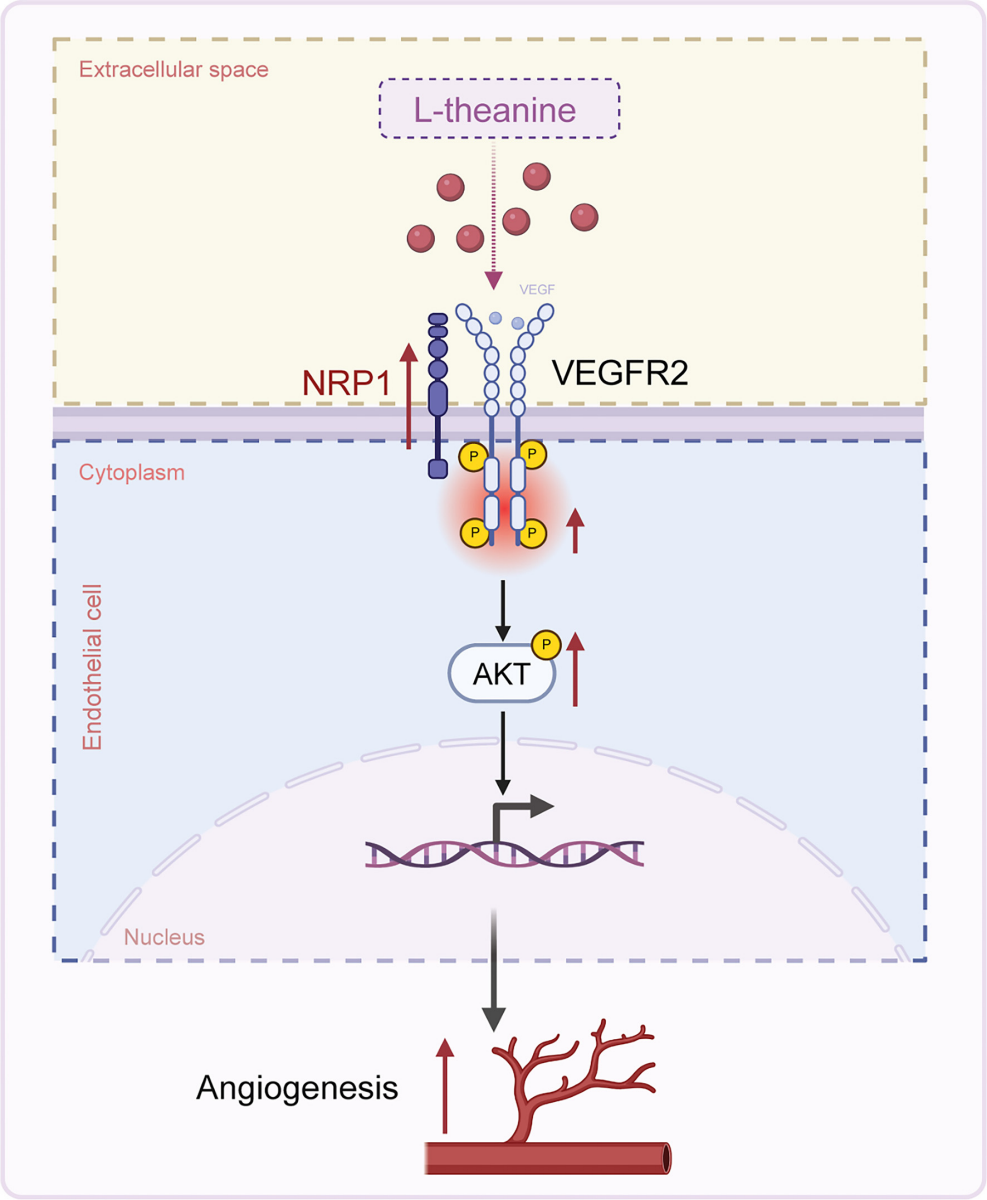
**L-theanine promotes angiogenesis through the modulation of the NRP1/VEGFR2/AKT pathway in ischemic conditions**. NRP1: Neuropilin-1; VEGFR2: Vascular endothelial growth factor receptor 2.

To further investigate the role of NRP1 in L-theanine-mediated angiogenesis, we performed HLI surgery on mice with NRP1 knockdown, achieved through adeno-associated virus injection (AAV-shNRP1), as well as on control mice (AAV-NC). L-theanine or PBS was administered intraperitoneally to these mice. Fourteen days post-surgery, mice with NRP1 silencing exhibited significantly lower blood flow recovery, even with L-theanine treatment (Figure 6A and 6B). Similarly, CD31 staining revealed that the L-theanine-induced increase in microvessel density was markedly reduced in NRP1-silenced mice (Figure 6C and 6D). These findings demonstrate that L-theanine enhances angiogenesis under ischemic conditions by positively regulating NRP1 (Figure 7).

## Discussion

The present study provides novel evidence supporting the positive effects of L-theanine on endothelial cells in promoting angiogenesis under ischemic conditions. Our data demonstrate that L-theanine significantly enhances tube formation, migration, sprouting, and proliferation of HUVECs *in vitro*. Furthermore, L-theanine treatment improved blood flow restoration and increased blood vessel density in a HLI mouse model. This study highlights the potential of L-theanine as a promising candidate for therapeutic strategies aimed at enhancing angiogenesis in ischemic conditions. PADs continue to pose significant global health challenges and are among the leading causes of mortality worldwide [39, 40]. Despite advancements in treatments, such as pharmacological therapies, interventional procedures, and surgical options, several persistent issues limit patient outcomes [41]. Traditional pharmacological treatments, including antiplatelet agents, anticoagulants, and lipid-lowering drugs, while effective in managing risk factors and preventing thrombotic events, often fail to sufficiently promote blood vessel formation in ischemic tissues [42–44]. A critical aspect of managing ischemic diseases is promoting angiogenesis—the process of forming new blood vessels—which is essential for restoring blood flow and facilitating tissue repair [45]. Our study introduces an innovative mechanism by which L-theanine modulates NRP1/VEGFR2 signaling to promote angiogenesis. Understanding this mechanism provides valuable insights into how L-theanine could be utilized to promote vascular regeneration in ischemic diseases. Moreover, given its ability to enhance blood flow restoration and increase blood vessel density in ischemic conditions, L-theanine shows potential as a therapeutic agent for conditions, such as PAD and myocardial ischemia. The proposed mechanism of action suggests that L-theanine could be used as a standalone therapy or in combination with existing treatments, thereby improving clinical outcomes.

L-theanine, a unique free amino acid, is a principal component of tea and a bioactive compound with numerous beneficial effects, including anti-inflammatory [46], antioxidant [21], and immune-regulatory properties [47]. The key finding of this study is the safety profile of L-theanine on HUVEC cells. Our experiments demonstrated that L-theanine, when administered at concentrations ranging from 0 to 1 mM for 48 h, did not negatively impact HUVEC cell viability. This finding suggests that L-theanine, a natural compound derived from tea, exhibits a strong safety profile at lower concentrations. However, at a higher concentration of 5 mM over the same duration, L-theanine impaired cell viability, indicating potential cytotoxic effects at elevated doses. These results suggest that while L-theanine may offer significant therapeutic benefits at lower concentrations, its potential adverse effects at higher concentrations warrant careful evaluation. The safety data provided by this study are critical for guiding the clinical application of L-theanine, emphasizing the importance of determining an appropriate dosage that balances therapeutic efficacy with minimal risk. Future research should focus on pharmacokinetic and pharmacodynamic studies to establish optimal dosing regimens and confirm the clinical relevance of L-theanine. This study provides valuable insights into the therapeutic potential of L-theanine, highlighting its promising safety profile at lower doses and underscoring the need for careful dosage considerations at higher concentrations.

Previous studies have found that in ear edema mouse models, L-theanine decreased the expression of platelet endothelial adhesion molecule-1 (PECAM-1) in endothelial cells [48]. However, the pro-angiogenic effects of L-theanine have not been fully explored. Our study addresses these gaps by demonstrating the potential of L-theanine to modulate NRP1/VEGFR2 signaling, offering a promising approach for clinical applications. L-theanine has been shown to exert protective effects on ischemic diseases, including cerebral ischemia and myocardial ischemia injury. Early research primarily focused on L-theanine’s potential to alleviate ischemic damage through antioxidant and anti-inflammatory mechanisms, particularly in the context of neurological protection [49, 50]. For instance, one study investigated the neuroprotective effects of L-theanine on ischemic brain damage in a mouse middle cerebral artery occlusion model, where L-theanine significantly reduced cerebral infarct size one day post-occlusion without affecting cerebral blood flow [50]. Another study indicated that L-theanine substantially reduced brain infarct size and improved neurological outcomes in a rat brain ischemia/reperfusion model [49]. Moreover, several studies have demonstrated L-theanine’s protective effects on myocardial ischemia/reperfusion injury (MIRI). A recent study reported that L-theanine reduced apoptosis and oxidative stress via the JAK2/STAT3 pathway, thereby mitigating MIRI-induced cardiac injury [51]. Another study revealed that L-theanine exerted cardioprotective effects against ischemia/reperfusion injury by inhibiting oxidative stress, preserving mitochondrial function, and upregulating antioxidant gene expression. These findings underscore the potential of L-theanine in ischemic diseases, particularly in its cardioprotective roles in cardiac ischemia [52]. Notably, our results revealed a novel pro-angiogenic effect of L-theanine in ischemic tissues, characterized by the upregulation of key angiogenic factors, such as NRP1 and VEGFR2. This upregulation enhanced endothelial cell proliferation, migration, and tube formation, which are pivotal processes for restoring blood flow and facilitating tissue repair. The mechanistic findings of our study demonstrated that L-theanine promoted angiogenesis by increasing the levels of NRP1 and VEGFR2, leading to activation of the AKT signaling pathway. However, further research is needed to elucidate the mechanisms by which L-theanine modulates NRP1 expression and to explore additional downstream pathways regulated by L-theanine via NRP1. Understanding these processes will provide deeper insights into L-theanine’s full mechanism of action and enhance its potential as a therapeutic agent. Our findings also indicated that the mRNA expression of NRP1 increased following L-theanine administration, suggesting multiple potential underlying mechanisms. The transcriptional regulation of NRP1 is a complex process influenced by various cellular signals and regulatory elements. While direct evidence of interactions between L-theanine and NRP1 transcription is limited, existing studies provide potential hypotheses. For example, we found that romidepsin, an inhibitor of histone deacetylases 1 and 2 (HDAC1 and HDAC2), may alter NRP1 gene expression [53]. Additionally, other studies suggest that NRP1 expression is influenced by regulatory factors, such as NUPR1, YAP1, NF-κB, and HIF1α [54–58]. Although these findings offer hypotheses for how L-theanine may affect NRP1 transcription, they remain speculative in the absence of experimental proof. Further research is necessary to identify the specific molecular interactions and signaling pathways involved.

Our results demonstrated that L-theanine promotes ischemic hindlimb angiogenesis and enhances the tube formation, migration, and proliferation of HUVECs. This effect is mediated through the activation of the NRP1/VEGFR2 signaling pathway. Although we did not directly quantify changes in VEGF levels, we observed a significant upregulation of NRP1 and VEGFR2 expression following L-theanine stimulation, suggesting an influence on VEGF signaling. Previous studies have highlighted the critical roles of NRP1 and VEGFR2 in processes, such as embryogenesis, wound healing, and the pathogenesis of various diseases [12]. VEGF-A binds to multiple receptors and co-receptors, including VEGFR2 and NRP1. Acting as a co-receptor with high affinity for VEGF, NRP1 enhances the activation of VEGFR2. This potentiation modulates VEGF-mediated signaling, which is crucial for endothelial cell migration, survival, and three-dimensional sprouting [38]. VEGFR2, predominantly expressed in vascular endothelial cells, functions as a major signal transducer for angiogenesis. By binding and activating VEGFR2, VEGF promotes endothelial cell proliferation, invasion, migration, survival, and increased vascular permeability and neovascularization [59]. Ligand binding to VEGFR2 induces conformational changes in its transmembrane domain, leading to increased phosphorylation of its kinase domain and subsequent activation [60]. Additionally, VEGF-A stimulates VEGFR2 kinase activity, triggering the activation of downstream signaling enzymes, such as PI3K and Akt, which are essential for cellular processes linked to angiogenesis, including survival, migration, and tube formation [61]. Consistent with this, our findings revealed that L-theanine increases VEGFR2/AKT phosphorylation levels in HUVECs, thereby contributing to angiogenesis. Despite these promising findings, several limitations of our study should be noted. First, we evaluated the effects of L-theanine using a mouse HLI model relevant to PAD, but species differences in vascular biology may limit the translational potential of our results. Additional validation in other ischemic models, such as myocardial infarction or clinical human trials, is necessary to confirm its therapeutic potential for PAD. Second, while we identified NRP1/VEGFR2 signaling as a key mediator of L-theanine’s pro-angiogenic effects, the precise molecular mechanisms regulating NRP1 expression in response to L-theanine remain unclear. Third, as a natural amino acid with diverse targets and functions, L-theanine may exert off-target or systemic effects. Our study focused solely on its pro-angiogenic properties, leaving potential interactions and systemic effects in PAD patients unexplored. Furthermore, we conducted experiments exclusively on HUVECs, which could limit the generalizability of our findings. Different endothelial cell lines or tissue-specific endothelial cells may have distinct receptor profiles or intracellular signaling pathways that could influence L-theanine’s effects on angiogenesis. Additionally, our study lacked comprehensive dose-response investigations. While we selected a concentration of 1 mM L-theanine for *in vitro* experiments based on initial viability assays, constructing a detailed dose–response curve would have provided greater insight into the optimal concentration and dose-dependent effects. This limitation may affect the accuracy of our conclusions regarding the most effective dose of L-theanine. In conclusion, our study identifies L-theanine as a promising candidate for the treatment of ischemic diseases, demonstrating a novel mechanism of action through modulation of the NRP1/VEGFR2 pathway. The natural origin and favorable safety profile of L-theanine, combined with its ability to effectively promote angiogenesis, position it as a potential therapeutic agent for further investigation in ischemic disease treatment.

## Supplemental data

**Table S1:** List of chemicals and reagents

**Chemicals and reagents**	**Source**
VEGF recombinant mouse protein	Cat# PMG0114 (Life Technologies)
Matrigel matrix (GFR)	Cat# 354230 (Corning)
Endothelial cell growth medium 2	Cat# C-22011 (Sigma-Aldrich)
Endothelial cell medium	Cat# 1001 (ScienCell)
L-theanine (98% purity)	Cat# SMB00395 (Sigma-Aldrich)
HUVEC	Cat# PCS-100-013(ATCC)

HUVEC: Human umbilical vein endothelial cell.

**Table S2:** List of the antibodies used for Western blots

**Antibodies**	**Source**	**Dilution**
CD31	550274 (BD, US)	1:1000
NRP1	ET1609-69 (HUABIO, China)	1:100
t-VEGFR2	2478S (CST, US)	1:500
p-VEGFR2 (1175)	2479S (CST, US)	1:1000
t-AKT	4691S (CST, US)	1:1000
p-AKT	4060S (CST, US)	1:1000
ACTIN	KC-5A08 (KANGCHEN, Shanghai, China)	1:5000

NRP1: Neuropilin-1; VEGFR2: Vascular endothelial growth factor receptor 2.

**Table S3:** List of the primer sequences used for qRT-PCR

**Gene**	**Species**	**Primers**
*ID1*	Homo sapiens	Forward: GCACGTCATCGACTACATCAG Reverse: ACGCATGCCGCCTCG
*RCAN1*	Homo sapiens	Forward: GTATGAATTGCACGCAGCGA Reverse: CGGCCTCCTGGTCTGGATA
*IRS1*	Homo sapiens	Forward: ACTTGAGCTACGGTGACGTG Reverse: AGCTGATGGTCTTGCTGGTC
*PRKCD*	Homo sapiens	Forward: TGACACTTGCCGCAGAGAAT Reverse: GGTAGAGTTCAAAGCGGCCT
*NRP1*	Homo sapiens	Forward: TGATGAAACAGGGAGCACGC Reverse: TGGTGATGAGGATGGGGTCT
*TFPI2*	Homo sapiens	Forward: GAGATCTGTCTCCTGCCCCT Reverse: TAGAAATTGTTGGCGTTGCCC
*LPAR1*	Homo sapiens	Forward: CTCGGCATAGTTCTGGACCC Reverse: CTGTGGACAGCACACGTCTA
*EPOR*	Homo sapiens	Forward: TACCCCACCCCACCTAAAGT Reverse: CATCGGATAAGCCCCCTTGG
*MMP15*	Homo sapiens	Forward: CTAAAGGGGCCTTCCTGAGC Reverse: GCAGGATGGACTTGGGGTAG
*CDC25C*	Homo sapiens	Forward: ACCTGCTCCTGGAGAGAGAC Reverse: GCAACGTTTTGGGGTTCCTC
*SOX4*	Homo sapiens	Forward: ACCTGAACCCCAGCTCAAAC Reverse: CAGTAGTCCGGGAACTCGAAG
*HDAC9*	Homo sapiens	Forward: AGCCTGACCTCATGTGGAAC Reverse: CTGTGCATTCTTTGCTGAGCC
*β-ACTIN*	Homo sapiens	Forward: ACGTTGCTATCCAGGCTGTG Reverse: GAGGGCATACCCCTCGTAGA

qRT-PCR: Quantitative RT-PCR.

## Data Availability

All gels, and microscopy images data from this study were packaged and submitted together in a zip package (https://zenodo.org/records/14461901). Please contact the corresponding authors for any other raw data needs. We guarantee that all raw data are available.

## References

[1] Criqui MH, Aboyans V (2015). Epidemiology of peripheral artery disease. Circ Res.

[2] Hirsch AT, Duval S (2013). The global pandemic of peripheral artery disease. Lancet.

[3] Conte MS, Bradbury AW, Kolh P, White JV, Dick F, Fitridge R (2019). Global vascular guidelines on the management of chronic limb-threatening ischemia. J Vasc Surg.

[4] Signorelli SS, Scuto S, Marino E, Xourafa A, Gaudio A (2019). Oxidative stress in peripheral arterial disease (PAD) mechanism and biomarkers. Antioxidants (Basel).

[5] Han J, Luo L, Marcelina O, Kasim V, Wu S (2022). Therapeutic angiogenesis-based strategy for peripheral artery disease. Theranostics.

[6] Dopheide JF, Geissler P, Rubrech J, Trumpp A, Zeller GC, Daiber A (2016). Influence of exercise training on proangiogenic TIE-2 monocytes and circulating angiogenic cells in patients with peripheral arterial disease. Clin Res Cardiol.

[7] McDermott MM (2018). Medical management of functional impairment in peripheral artery disease: a review. Prog Cardiovasc Dis.

[8] Willems LH, Maas D, Kramers K, Reijnen M, Riksen NP, Ten Cate H (2022). Antithrombotic therapy for symptomatic peripheral arterial disease: a systematic review and network meta-analysis. Drugs.

[9] Golledge J (2022). Update on the pathophysiology and medical treatment of peripheral artery disease. Nat Rev Cardiol.

[10] Hwang D, Park SH, Koo BK (2023). Ischemia with nonobstructive coronary artery disease: concept, assessment, and management. JACC Asia.

[11] Carmeliet P (2003). Angiogenesis in health and disease. Nat Med.

[12] Carmeliet P, Jain RK (2011). Molecular mechanisms and clinical applications of angiogenesis. Nature.

[13] Turkozu D, Sanlier N (2017). L-theanine, unique amino acid of tea, and its metabolism, health effects, and safety. Crit Rev Food Sci Nutr.

[14] Xu L, Han F, Zhang X, Yu Q (2020). Ultrasound enhanced biosynthesis of L-theanine from L-glutamine and ethylamine by recombinant gamma-glutamyltranspeptidase. Bioresour Technol.

[15] Li MY, Liu HY, Wu DT, Kenaan A, Geng F, Li HB (2022). L-theanine: a unique functional amino acid in tea (Camellia sinensis L.) with multiple health benefits and food applications. Front Nutr.

[16] Liu A, Lin L, Xu W, Gong Z, Liu Z, Xiao W (2021). L-theanine regulates glutamine metabolism and immune function by binding to cannabinoid receptor 1. Food Funct.

[17] Mu W, Zhang T, Jiang B (2015). An overview of biological production of L-theanine. Biotechnol Adv.

[18] Chen S, Kang J, Zhu H, Wang K, Han Z, Wang L (2023). L-theanine and immunity: a review. Molecules.

[19] Hidese S, Ogawa S, Ota M, Ishida I, Yasukawa Z, Ozeki M (2019). Effects of L-theanine administration on stress-related symptoms and cognitive functions in healthy adults: a randomized controlled trial. Nutrients.

[20] Mancini E, Beglinger C, Drewe J, Zanchi D, Lang UE, Borgwardt S (2017). Green tea effects on cognition, mood and human brain function: a systematic review. Phytomedicine.

[21] Paiva L, Lima E, Motta M, Marcone M, Baptista J (2020). Variability of antioxidant properties, catechins, caffeine, L-theanine and other amino acids in different plant parts of Azorean Camellia sinensis. Curr Res Food Sci.

[22] Raimondi C, Brash JT, Fantin A, Ruhrberg C (2016). NRP1 function and targeting in neurovascular development and eye disease. Prog Retin Eye Res.

[23] Liu ZL, Chen HH, Zheng LL, Sun LP, Shi L (2023). Angiogenic signaling pathways and anti-angiogenic therapy for cancer. Signal Transduct Target Ther.

[24] Potente M, Gerhardt H, Carmeliet P (2011). Basic and therapeutic aspects of angiogenesis. Cell.

[25] Wen HZ, Xie P, Zhang F, Ma Y, Li YL, Xu SK (2018). Neuropilin 1 ameliorates electrical remodeling at infarct border zones in rats after myocardial infarction. Auton Neurosci.

[26] Volpert OV, Pili R, Sikder HA, Nelius T, Zaichuk T, Morris C (2002). Id1 regulates angiogenesis through transcriptional repression of thrombospondin-1. Cancer Cell.

[27] Jin H, Wang C, Jin G, Ruan H, Gu D, Wei L (2017). Regulator of calcineurin 1 gene isoform 4, down-regulated in hepatocellular carcinoma, prevents proliferation, migration, and invasive activity of cancer cells and metastasis of orthotopic tumors by inhibiting nuclear translocation of NFAT1. Gastroenterology.

[28] Wang Y, Zhang X, Zou C, Kung HF, Lin MC, Dress A (2016). miR-195 inhibits tumor growth and angiogenesis through modulating IRS1 in breast cancer. Biomed Pharmacother.

[29] Lizotte F, Pare M, Denhez B, Leitges M, Guay A, Geraldes P (2013). PKCdelta impaired vessel formation and angiogenic factor expression in diabetic ischemic limbs. Diabetes.

[30] Mo J, Zhao X, Wang W, Zhao N, Dong X, Zhang Y (2021). TFPI2 promotes perivascular migration in an angiotropism model of melanoma. Front Oncol.

[31] Lin YH, Lin YC, Chen CC (2021). Lysophosphatidic acid receptor antagonists and cancer: the current trends, clinical implications, and trials. Cells.

[32] Zhang M, Sui W, Cheng C, Xue F, Tian Z, Cheng J (2021). Erythropoietin promotes abdominal aortic aneurysms in mice through angiogenesis and inflammatory infiltration. Sci Transl Med.

[33] Wang Y, Zhang L, Wei N, Sun Y, Pan W, Chen Y (2020). Silencing LINC00482 inhibits tumor-associated inflammation and angiogenesis through down-regulation of MMP-15 via FOXA1 in bladder cancer. Aging (Albany NY).

[34] Tomooka F, Kaji K, Nishimura N, Kubo T, Iwai S, Shibamoto A (2023). Sulforaphane potentiates gemcitabine-mediated anti-cancer effects against intrahepatic cholangiocarcinoma by inhibiting HDAC activity. Cells.

[35] Aaboe M, Birkenkamp-Demtroder K, Wiuf C, Sorensen FB, Thykjaer T, Sauter G (2006). SOX4 expression in bladder carcinoma: clinical aspects and in vitro functional characterization. Cancer Res.

[36] Lan Z, Chen A, Li L, Ye Y, Liang Q, Dong Q (2022). Downregulation of HDAC9 by the ketone metabolite beta-hydroxybutyrate suppresses vascular calcification. J Pathol.

[37] Herzog B, Pellet-Many C, Britton G, Hartzoulakis B, Zachary IC (2011). VEGF binding to NRP1 is essential for VEGF stimulation of endothelial cell migration, complex formation between NRP1 and VEGFR2, and signaling via FAK Tyr407 phosphorylation. Mol Biol Cell.

[38] Sharma S, Ehrlich M, Zhang M, Blobe GC, Henis YI (2024). NRP1 interacts with endoglin and VEGFR2 to modulate VEGF signaling and endothelial cell sprouting. Commun Biol.

[39] Liu S, Li Y, Zeng X, Wang H, Yin P, Wang L (2019). Burden of cardiovascular diseases in China, 1990-2016: findings from the 2016 global burden of disease study. JAMA Cardiol.

[40] Wang W, Hu M, Liu H, Zhang X, Li H, Zhou F (2021). Global burden of disease study 2019 suggests that metabolic risk factors are the leading drivers of the burden of ischemic heart disease. Cell Metab.

[41] Hausenloy DJ, Yellon DM (2016). Ischaemic conditioning and reperfusion injury. Nat Rev Cardiol.

[42] Capodanno D, Mehran R, Krucoff MW, Baber U, Bhatt DL, Capranzano P (2023). Defining strategies of modulation of antiplatelet therapy in patients with coronary artery disease: a consensus document from the academic research consortium. Circulation.

[43] Pierno S, Musumeci O (2023). Pharmacotherapy of the lipid-lowering drugs: update on efficacy and risk. Int J Mol Sci.

[44] Pasqualetti G, Danesi R, Del Tacca M, Bocci G (2007). Vascular endothelial growth factor pharmacogenetics: a new perspective for anti-angiogenic therapy. Pharmacogenomics.

[45] Eelen G, Treps L, Li X, Carmeliet P (2020). Basic and therapeutic aspects of angiogenesis updated. Circ Res.

[46] Bai H, Zhang Z, Li Y, Song X, Ma T, Liu C (2020). L-theanine reduced the development of knee osteoarthritis in rats via its anti-inflammation and anti-matrix degradation actions: in vivo and in vitro study. Nutrients.

[47] Zhao J, Zhao X, Tian J, Xue R, Luo B, Lv J (2020). Theanine attenuates hippocampus damage of rat cerebral ischemia-reperfusion injury by inhibiting HO-1 expression and activating ERK1/2 pathway. Life Sci.

[48] Zeng WJ, Tan Z, Lai XF, Xu YN, Mai CL, Zhang J (2018). Topical delivery of l-theanine ameliorates TPA-induced acute skin inflammation via downregulating endothelial PECAM-1 and neutrophil infiltration and activation. Chem Biol Interact.

[49] Zukhurova M, Prosvirnina M, Daineko A, Simanenkova A, Petrishchev N, Sonin D (2013). L-theanine administration results in neuroprotection and prevents glutamate receptor agonist-mediated injury in the rat model of cerebral ischemia-reperfusion. Phytother Res.

[50] Egashira N, Hayakawa K, Mishima K, Kimura H, Iwasaki K, Fujiwara M (2004). Neuroprotective effect of gamma-glutamylethylamide (theanine) on cerebral infarction in mice. Neurosci Lett.

[51] Li Q, Ding J, Xia B, Liu K, Zheng K, Wu J (2024). L-theanine alleviates myocardial ischemia/reperfusion injury by suppressing oxidative stress and apoptosis through activation of the JAK2/STAT3 pathway in mice. Mol Med.

[52] Garcia-Nino WR, Correa F, Zuniga-Munoz AM, Jose-Rodriguez A, Castaneda-Gomez P, Mejia-Diaz E (2024). L-theanine abates oxidative stress and mitochondrial dysfunction in myocardial ischemia-reperfusion injury by positively regulating the antioxidant response. Toxicol Appl Pharmacol.

[53] Nerlakanti N, McGuire JJ, Bishop RT, Nasr MM, Li T, Reed DR (2024). Histone deacetylase upregulation of neuropilin-1 in osteosarcoma is essential for pulmonary metastasis. Cancer Lett.

[54] Dong Y, Ma WM, Shi ZD, Zhang ZG, Zhou JH, Li Y (2021). Role of NRP1 in bladder cancer pathogenesis and progression. Front Oncol.

[55] Zhu Q, Li J, Wu Q, Cheng Y, Zheng H, Zhan T (2020). Linc-OIP5 in the breast cancer cells regulates angiogenesis of human umbilical vein endothelial cells through YAP1/Notch/NRP1 signaling circuit at a tumor microenvironment. Biol Res.

[56] Fu R, Du W, Ding Z, Wang Y, Li Y, Zhu J (2021). HIF-1alpha promoted vasculogenic mimicry formation in lung adenocarcinoma through NRP1 upregulation in the hypoxic tumor microenvironment. Cell Death Dis.

[57] Dai X, Okon I, Liu Z, Wu Y, Zhu H, Song P (2017). A novel role for myeloid cell-specific neuropilin 1 in mitigating sepsis. FASEB J.

[58] Matilla L, Arrieta V, Jover E, Garcia-Pena A, Martinez-Martinez E, Sadaba R (2020). Soluble St2 induces cardiac fibroblast activation and collagen synthesis via neuropilin-1. Cells.

[59] Modi SJ, Kulkarni VM (2019). Vascular endothelial growth factor receptor (VEGFR-2)/KDR inhibitors: medicinal chemistry perspective. Med. Drug Discov.

[60] Sarabipour S, Ballmer-Hofer K, Hristova K (2016). VEGFR-2 conformational switch in response to ligand binding. Elife.

[61] Ruan GX, Kazlauskas A (2012). Axl is essential for VEGF-A-dependent activation of PI3K/Akt. EMBO J.

